# Evaluating Neural Radiance Fields for 3D Plant Geometry Reconstruction in Field Conditions

**DOI:** 10.34133/plantphenomics.0235

**Published:** 2024-09-09

**Authors:** Muhammad Arbab Arshad, Talukder Jubery, James Afful, Anushrut Jignasu, Aditya Balu, Baskar Ganapathysubramanian, Soumik Sarkar, Adarsh Krishnamurthy

**Affiliations:** ^1^ Department of Computer Science, Iowa State University, Ames, IA, USA.; ^2^ Department of Mechanical Engineering, Iowa State University, Ames, IA, USA.

## Abstract

We evaluate different Neural Radiance Field (NeRF) techniques for the 3D reconstruction of plants in varied environments, from indoor settings to outdoor fields. Traditional methods usually fail to capture the complex geometric details of plants, which is crucial for phenotyping and breeding studies. We evaluate the reconstruction fidelity of NeRFs in 3 scenarios with increasing complexity and compare the results with the point cloud obtained using light detection and ranging as ground truth. In the most realistic field scenario, the NeRF models achieve a 74.6% F1 score after 30 min of training on the graphics processing unit, highlighting the efficacy of NeRFs for 3D reconstruction in challenging environments. Additionally, we propose an early stopping technique for NeRF training that almost halves the training time while achieving only a reduction of 7.4% in the average F1 score. This optimization process substantially enhances the speed and efficiency of 3D reconstruction using NeRFs. Our findings demonstrate the potential of NeRFs in detailed and realistic 3D plant reconstruction and suggest practical approaches for enhancing the speed and efficiency of NeRFs in the 3D reconstruction process.

## Introduction

In recent years, reconstructing 3-dimensional (3D) geometry has emerged as a critical area within plant sciences. As global challenges in food production become increasingly complex [1], gaining a detailed understanding of plant structures has become essential. This goes beyond mere visual representation; capturing the intricate details of plant geometry provides valuable insights into their growth, responses to environmental stressors, and physiological processes [2,3]. Consequently, there are several efforts for the 3D reconstruction of plants [4–6].

One of the most common approaches for 3D reconstruction is photogrammetry, which relies on the analysis of discrete 2D pixels using techniques such as structure from motion (SfM) [7] and multiview stereo [8]. Another direct approach is utilizing light detection and ranging (LiDAR) scanners (such as FARO 3D LiDAR scanner) to capture a dense 3D point cloud of the plants. This approach has been successfully used for the 3D reconstruction of maize [9] and tomato plants [10]. It is challenging for contemporary 3D modeling techniques to capture the minute details inherent in plant structures [2]. The complexity of plants, from their delicate leaf venation [11] to intricate branching patterns [12], necessitates models that encompass these specific details. Scans from multiple angles are essential to capture every detail, which is challenging since multiple LiDAR scans are time consuming. Due to the limited poses, this approach does not scale well to capture minute details in large scenes; consequently, some desired details might be missed in the final model. Andújar et al. [13] have emphasized that, even with advanced sensors, there are gaps in detailed reconstruction. They also point out that while devices such as the MultiSense S7 from Carnegie Robotics combine lasers, depth cameras, and stereo vision to offer reasonable results, the high acquisition costs can be prohibitive. At the same time, while photogrammetry is adept at large-scale reconstructions, it often cannot capture subtle details of plants [9,10,14].

In addition to the challenges mentioned above, the dynamic nature of flexible objects such as plants and their environment introduces an added complexity. Plants, unlike static entities, undergo growth, exhibit movement in reaction to environmental stimuli such as wind, and demonstrate both diurnal and seasonal variations. The environmental dynamism, coupled with plant behavior, further complicates modeling efforts. The comprehensive investigation of Paturkar et al. [14] underscores that this dynamism inherently complicates the attainment of precise 3D models. Factors such as persistent growth, environmental dynamism, and external perturbations, notably in windy scenarios, jeopardize the consistency of data acquisition during imaging processes [15,16]. Liénard et al. [17] highlight that errors in postprocessing unmanned aerial vehicle-based 3D reconstructions can lead to severe, irreversible consequences. This complexity necessitates innovative solutions in 3D modeling and data processing.

**Fig. 1.**
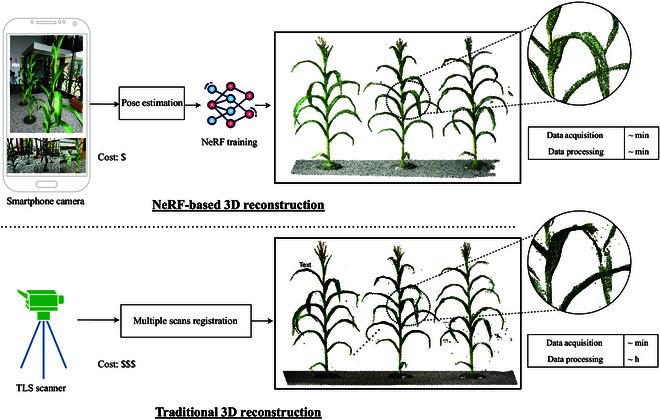
NeRFs are proposed as an alternative to traditional TLS scans for 3D plant reconstruction, offering cost-effective and efficient modeling from images captured at multiple angles using a smartphone camera, in contrast to the higher expense and extensive processing time required by TLS for multiangle scan registration.

One of the most recent approaches for 3D reconstruction is Neural Radiance Fields (NeRFs). At its core, NeRFs utilize deep learning to synthesize continuous 3D scenes by modeling the complete volumetric radiance field [18]. NeRFs enable the rendering of photorealistic scenes from any viewpoint from a neural network trained using a set of 2D images without necessitating explicit 3D geometry or depth maps. NeRFs use implicit representations of the volumetric scene, in contrast to explicit representations such as point clouds in SfM and voxel grids in multiview stereo. The implicit representation utilized by NeRF is resolution-invariant, allowing for more detailed and granular modeling without the constraints of resolution-dependent methods. The versatility and rapid adoption of NeRF as a state-of-the-art technique in computer vision and graphics underscore its relevance, with applications ranging from virtual reality [19] to architectural reconstructions [20]. Particularly in plant science research, NeRF’s ability to capture fine details offers the potential for deep insights into plant structures and has the potential to be a vital tool in plant phenotyping and breeding (see Fig. 1).

These factors indicate that the challenges in capturing detailed plant structures remain, even when employing sophisticated sensors. Financial implications further exacerbate these challenges. Traditional 3D modeling techniques often fall short of accurately capturing the complex 3D structures of plants [21]. Although direct techniques such as LiDAR scanners provide better accuracy, their exorbitant costs often render them inaccessible to many researchers. Tang et al. [22] delineate that the financial commitment associated with such advanced equipment, combined with the specialized expertise requisite for its operation, limits their adoption within academic and enthusiast domains.

In this paper, we perform a detailed evaluation of NeRF methodologies to assess their applicability and effectiveness for high-resolution 3D reconstruction of plant structures. An essential part of our study involves a comparative analysis of different NeRF implementations to determine the most effective framework for specific plant modeling needs. This includes assessing the methods’ fidelity, computational efficiency, and ability to adapt to changes in environmental conditions. Such comparative analysis is crucial for establishing benchmarks for NeRF’s current capabilities and identifying future technological improvement opportunities. Building on this foundation, we introduce an early stopping algorithm to preemptively terminate the training process, substantially reducing computational cost while retaining model precision. We summarize our contributions as follows:

1. A dataset collection encompassing a wide range of plant scenarios for reconstruction purposes consisting of images, camera poses, and ground truth terrestrial laser scanning (TLS) scans.

2. An evaluation of state-of-the-art NeRF techniques across different 2D and 3D metrics, offering insights for further research.

3. An early stopping algorithm to efficiently halt the NeRF training when improvements in model fidelity no longer justify computational costs, ensuring optimal resource use.

4. The development of an end-to-end 3D reconstruction framework using NeRFs designed specifically for the 3D reconstruction of plants.

Our research aims to explore the feasibility of NeRFs for the 3D reconstruction of plants offering an in-depth analysis. A pivotal aspect of our methodology is using low-cost mobile cameras for data acquisition. By utilizing the widespread availability and imaging capabilities of modern smartphones, we can make high-quality image data collection more accessible and cost-effective. This approach, combined with the NeRFs’ ability to process various image datasets for 3D reconstruction, can revolutionize plant reconstruction efforts.

The rest of the paper is arranged as follows. In Materials and Methods, we outline the dataset collection, NeRF implementations, evaluation methods, and the Learned Perceptual Image Patch Similarity (LPIPS)-based early stopping algorithm. In Results, we analyze results from single and multiple plant scenarios, both indoors and outdoors, using critical performance metrics. Finally, in Discussion, we provide a theoretical discussion on the sampling strategies of different NeRF implementations and examine their impact on performance.

## Materials and Methods

**Fig. 2.**
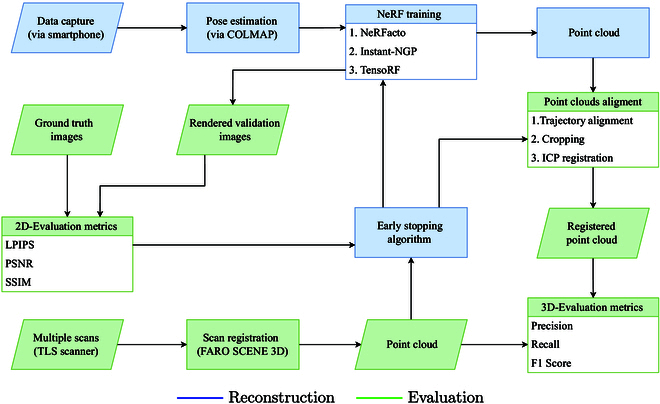
Workflow for 3D reconstruction and evaluation. The different steps of the above workflow is explained in detail below.

To evaluate 3D plant reconstruction using NeRFs, we propose a comprehensive methodology encompassing data collection, NeRF implementations, evaluation metrics, and an early stopping algorithm. The overall workflow of the different steps of our framework is shown in Fig. 2.

### Evaluation scenarios and data collection

**Fig. 3.**
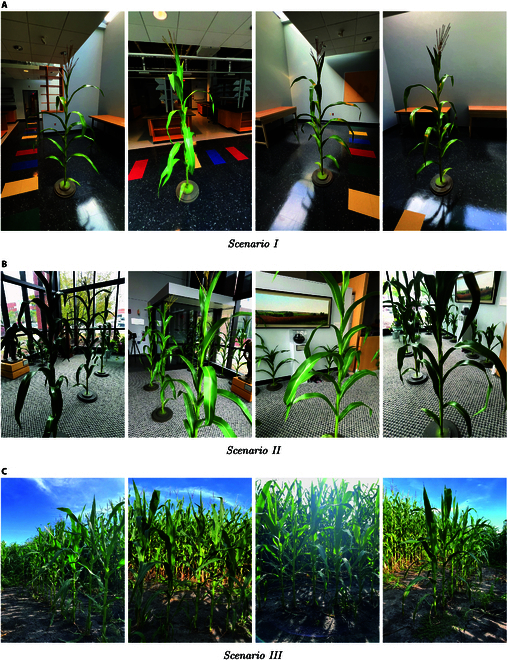
Example images input to NeRFs for reconstruction across 3 different scenarios. (A) Scenario I: Indoor single object. (B) Scenario II: Indoor multiple objects. (C) Scenario III: Outdoor scene.

We evaluate NeRFs, examining 3 distinct scenarios with ground truth data, from controlled indoor to dynamic outdoor environments, and a final testing scenario. The 4 scenarios are:

1. Single Corn Plant Indoor: This serves as the simplest test case. A solitary corn plant is placed in a controlled indoor environment. The lighting, background, and other environmental factors are kept constant. The objective is to assess the basic capabilities of NeRF in reconstructing an individual plant structure [23] (see Fig. 3A).

2. Multiple Corn Plants Indoor: In this case, more than one corn plant is situated in an indoor setting. The increased complexity due to multiple plants poses a greater challenge for the 3D reconstruction. Interplant occlusions and varying plant orientations add an additional layer of complexity (see Fig. 3B).

3. Multiple Corn Plants in a Field with Other Plants: This scenario represents a real-world agricultural field, where corn plants are interspersed with other types of plants. The added complexity due to variable lighting, wind, and other dynamic environmental conditions tests the robustness of the NeRF technology (see Fig. 3C). We selected a row plot of corn plants planted at approximately 0.2-m distance, approximately at the V12 stage. The leaves between 2 neighboring plants are overlapping.

4. In-field Test Data: For validating the proposed early stopping methodology, a diverse dataset was assembled, featuring scenarios with soybean, *Anthurium* *hookeri*, a mixture of plants, *Cymbidium* *floribundum*, and *Hydrangea* *paniculata*.

Our training dataset for NeRF is sourced from red-green-blue (RGB) images and LiDAR data captured using a mobile phone, with the RGB images aiding in the 3D reconstruction of the plants and the LiDAR exclusively for pose capture. For all 3 scenarios, data is captured using an iPhone 13 Pro featuring 4K resolution. The device is held at a constant height while circling the plant to ensure consistent capture angles. The data collection process utilizes the Polycam app [24], with approximately 2.5 min for Scenario III (multiple plants in the outdoor setting) and around 1 min for Scenario I (single plant in the indoor setting). To establish accurate ground truth, we utilized high-definition terrestrial LiDAR scans using the Faro Focus S350 Scanner. The scanner has an angular resolution of 0.011°, equating to a 1.5-mm point spacing over a 10-m scanning range, and the capacity to acquire point clouds of up to 700 million points at 1 million points per second. Additionally, the scanner includes a built-in RGB camera that captures 360° images once the scanning process is complete.

Both in indoor and outdoor settings, we scan the plants from 4 (for the single plant) to 6 (for multiple plants) locations around the plant(s) at a height of 1.5 m and a distance of 1.5 m from the plant(s). To reduce the movement of the leaves during scanning, in indoor settings, we ensure that there is no airflow around the plants, and in outdoor settings, we waited for a suitable time when there was negligible wind flow (2023 August 31, at 8:30 AM). Each scan required approximately 2.5 min, totaling a capture time of around 18 min in outdoor settings, including manually moving the scanner around the plot. The 6 scans were processed in SCENE software to add RGB color data to the point clouds, followed by the registration of the clouds by minimizing cloud-to-cloud distance and top view distance. Afterward, we cropped out the area of interest from the registered point cloud, removed duplicate points, and reduced noise using statistical outlier removal based on global and local point-to-point distance distributions. This process resulted in the point cloud having an average resolution of about 7 mm. This experimental setup enables the NeRF algorithm to work on a range of complexities, from controlled environments to dynamic, real-world conditions.

Camera pose estimation is a crucial second step, typically achieved through an SfM pipeline such as COLMAP [25]. This process is essential for obtaining accurate 3D structures from sequences of images by determining correspondences between feature points and by using sequential matching, especially effective since our dataset comprises video frames.

### NeRFs

NeRFs model a scene as a continuous function mapping a 3D position **x** = (*x*, *y*, *z*) and a 2D viewing direction **d** = (*θ*, *ϕ*) to a color **c** = (*r*, *g*, *b*) and density *σ*. The function is parameterized by a neural network *F_θ_*, expressed as:

$$\left(c,\sigma \right)=F_{\theta }\left(x,d\right)$$ (1)

Rendering an image involves integrating the color and density along camera rays, a process formalized as:

$$C\left(r\right)=\int_{t_{n}}^{t_{f}}‍T\left(t\right)\sigma \left(r\left(t\right)\right)c\left(r\left(t\right),d\right)\mathrm{dt}$$ (2)

where $T\left(t\right)=\text{exp}\left(-\int_{t_{n}}^{t}‍\sigma \left(r\left(s\right)\right)\mathrm{ds}\right)$ represents the accumulated transmittance along the ray **r**(*t*) = **o** + *t***d**, with **o** being the ray origin and [*t_n_*, *t_f_*] the near and far bounds. In our workflow, we incorporate some of the state-of-the-art NeRF implementations optimized for their 3D reconstruction capabilities, which are critical to enable large-scale plant phenotyping studies. Specifically, we employ Instant-NGP [26], TensoRF [27], and NeRFacto [28].

We specifically chose Instant-NGP, TensoRF, and NeRFacto to evaluate for plant reconstruction since these implementations are more efficient and achieve comparable results as a vanilla NeRF approximately 50 times faster. Each of these implementations introduces several new features over the vanilla NeRF implementations. Instant-NGP introduces a small neural network complemented by a multiresolution hash table, optimizing the number of operations required for training and rendering [26]. TensoRF, on the other hand, conceptualizes the radiance field as a 4D tensor and applies tensor decomposition to achieve better rendering quality and faster reconstruction times compared to the traditional NeRF approach [27]. NeRFacto combines various techniques such as the Multilayer Perceptron (MLP) adapted from Instant-NGP and the Proposal Network Sampler from MipNeRF-360 [29]. Apart from these 3 methods, we also tried the vanilla Mip-NeRF [30]. Unfortunately, Mip-NeRF fails to reconstruct more complicated 3D scenes (such as Scenario II) in our testing. Please refer to the Supplementary Materials where we provide a table for training (over time) of MipNeRF. We briefly describe the 3 tested NeRF approaches below.

Instant-NGP: Instant-NGP introduces advancements in NeRFs by focusing on 3 key improvements: enhanced sampling through occupancy grids, a streamlined neural network architecture, and a multiresolution hash encoding technique. The hallmark of Instant-NGP is its multiresolution hash encoding. This approach maps input coordinates to trainable feature vectors stored across multiple resolutions. For each input coordinate, the method hashes surrounding voxel vertices, retrieves and interpolates the corresponding feature vectors, and then inputs these interpolated vectors into the neural network. This process enhances the model’s ability to learn complex geometries and ensures a smoother function due to the trainable nature of the feature vectors. The overall design of Instant-NGP drastically accelerates NeRF training and rendering, enabling near real-time processing capabilities. These enhancements collectively empower Instant-NGP to achieve speedups of up to 1,000×. The method also employs multiscale occupancy grids to efficiently bypass empty space and areas beyond dense media during sampling, thereby reducing the computational load. These occupancy grids are dynamically updated based on the evolving understanding of the scene’s geometry, facilitating an increase in sampling efficiency. In parallel, Instant-NGP adopts a compact, fully fused neural network architecture designed for rapid execution. This network is optimized to operate within a single CUDA kernel, consisting of only 4 layers with 64 neurons each, resulting in a speed boost—achieving a 5 to 10 times faster performance than traditional NeRF implementations.

TensoRF: TensoRF improves scene representation by modeling the radiance field as a 4D tensor within a 3D voxel grid, where each voxel is enriched with multichannel features. This model leverages tensor decomposition to efficiently manage the high-dimensional data, utilizing 2 key techniques: Canonic Polyadic (CP) and Vector-Matrix (VM) decompositions. CP decomposition simplifies the tensor into rank-one components using compact vectors, reducing the model’s memory footprint. VM decomposition, alternatively, breaks the tensor into compact vector and matrix factors, striking a balance between memory efficiency and detail capture. These enable TensoRF to reduce memory requirements while enhancing rendering quality and accelerating reconstruction times. CP decomposition leads to faster scene reconstruction with improved rendering quality and a smaller model size compared to conventional NeRF approaches. VM decomposition takes this further, offering even better rendering quality and quicker reconstruction, all within a compact model size.

NeRFacto: NeRFacto is an aggregate of techniques optimized for rendering static scenes from real images. The model enhances the NeRF framework by incorporating pose refinement and advanced sampling strategies to improve the fidelity of the scene reconstruction. Pose refinement is critical when initial camera poses are imprecise, which is often the case with mobile capture technologies. NeRFacto refines these poses, thus mitigating artifacts and enhancing detail. The model employs a piecewise sampler for initial scene sampling, allocating samples to optimize the coverage of both near and distant objects. This is further refined using a proposal sampler, which focuses on areas that contribute most to the scene’s appearance and is informed by a density function derived from a small, fused MLP with hash encoding. Such a design ensures efficient sampling and better reconstruction. Further explanation and contrast with Instant-NGP is given in the discussion section. The implementations for aforementioned algorithms are taken from the open-source project NeRFStudio [28].

There have been several recent works that have compared NeRF approaches for 3D reconstruction. Table 1 summarizes some recent work evaluating different NeRF methodologies. Some of these recent research works also employ additional methods to improve reconstruction fidelity. For example, SteerNeRF [31] utilizes neural sparse voxel fields (NSVFs) [32], KiloNeRF [33], PlenOctree [34], and DIVeR [35], to obtain a smooth rendering from different viewpoints. NSVF introduces a fast, high-quality, viewpoint-free rendering method using a sparse voxel octree for efficient scene representation. KiloNeRF accelerates NeRF’s rendering by 3 orders of magnitude using thousands of tiny MLPs, maintaining visual quality with efficient training. PlenOctree uses an Octree data structure to store the Plenoptic function. DIVeR improves upon NeRF by using deterministic estimates for volume rendering, allowing for realistic 3D rendering from few images. Similar to our work, Azzarelli et al. [36] propose a framework for evaluating NeRF methods using Instant-NGP, NeRFacto, and Mip-NeRF, focusing on neural rendering isolation and parametric evaluation. Radl et al. [37] analyze trained vanilla NeRFs, Instant-NGP, NeRFActo, and Mip-NeRF, showing accelerated computations by transforming activation features, reducing computations by 50%.

**Table 1.** Recent works comparing the performance of different NeRF techniques for 3D reconstruction applications

Paper	Instant-NGP	NeRFacto	TensoRF	NeRF	Additional methods
Azzarelli et al. [36]	✓ ^a^	✓ ^a^	×	×	Mip-NeRF
Radl et al. [37]	×	✓ ^a^	×	✓ ^a^	Mip-NeRF
Li et al. [31]	✓ ^a^	×	×	✓ ^b^	NSVF, PlenOctree, KiloNeRF, DIVeR
Remondino et al. [38]	✓ ^b^	✓ ^a^	✓ ^b^	×	MonoSDF, VolSDF, NeuS, UniSurf
Balloni et al. [43]	✓ ^b^	×	×	×	-
Ours	✓ ^a^	✓ ^a^	✓ ^a^	×	-

^a^ Used implementation in NeRFStudio or SDFStudio.

^b^ Used original implementation.

Remondino et al. [38] analyze image-based 3D reconstruction comparing different NeRFs (including Instant-NGP, NeRFacto, TensoRF, MonoSDF [39], VolSDF [40], NeUS [41], and UniSurf [42]) with traditional photogrammetry, highlighting their applicability and performance differences for reconstructing heritage scenes and monuments. Balloni et al. [43] does the same but with using only Instant-NGP. Each of these different NeRF implementations have some advancements over vanilla NeRF. MonoSDF demonstrates that incorporating monocular geometry cues improves the quality of neural implicit surface reconstruction. VolSDF improves the volume rendering of signed distance fields (SDF) using a new density representation. NeuS introduces a bias-free volume rendering method for neural surface reconstruction, outperforming existing techniques in handling complex structures and self-occlusions. UniSurf combines implicit surface models and radiance fields, enhancing 3D reconstruction and novel view synthesis without input masks.

### 3D registration

We reconstruct the scene and capture point clouds using a FARO scan for ground truth. 3D registration or alignment is crucial to perform a one-to-one comparison between the NeRF-based reconstruction and ground truth. Our alignment and evaluation methodology is adapted from Knapitsch et al. [44]. In their work, they evaluate different pipelines and use COLMAP as an “arbitrary reference” frame. However, in our case, all the NeRFs use COLMAP in their pipeline, so the reference and reconstruction frames become the same. The steps used for registration are:

Preliminary Camera Trajectory Alignment: The NeRF-reconstructed point cloud is manually aligned with the ground truth using point-based alignment. Four corresponding points are selected in both point clouds to compute an initial transformation matrix. This matrix aligns the camera poses, providing initial scale and orientation estimates. This initial coarse-grained alignment step paves the way for more detailed alignment procedures.

Cropping: Each ground truth model has a manually defined bounding volume, outlining the evaluation region for reconstruction.

Iterative Closest Point Registration: Drawing inspiration from the iterative refinement process detailed by Besl and McKay [45] and further refined by Zhang [46], we adopt a 3-stage approach [44] for our initial registration framework. The process begins with a specified voxel size and an associated threshold for the initial registration. In the next iteration, the transformation result from the previous step is used as a starting point, with the voxel size reduced by half to achieve finer detail in the registration. The third stage aims to refine the alignment further by returning to the original voxel size and adjusting the threshold to facilitate convergence at each stage. This multiscale strategy is designed to capture both coarse and fine details, thereby improving the accuracy and precision of the model alignment. However, in our adaptation for plant structure reconstruction, we diverged from Knapitsch et al. [44] by maintaining the iterative process within a single stage rather than expanding across multiple stages. We found that increasing the iteration count 10-fold, rather than the number of stages, prevented the registration process from collapsing [47].

### Evaluation metrics

To assess the similarity between the ground truth (obtained from TLS) and the reconstructed 3D point cloud, the following metrics are employed:

1. Precision/Accuracy. Given a reconstructed point set R and a ground truth set G, the precision metric *P*(*d*) assesses the proximity of points in R to G within a distance threshold *d*. Mathematically, it is formulated as:

$$P\left(d\right)=\frac{100}{|R|}\sum_{r\in R}‍I\left(\underset{g\in G}{\text{min}}∥\;r-g\;∥<d\right),$$ (3)

where $I\left(\cdot \right)$ is an indicator function. Precision values ranges from 0 to 100, with higher values indicating better performance.

2. Recall/Completeness. Conversely, the recall metric *R*(*d*) quantifies how well the reconstruction R encompasses the points in the ground truth G for a given distance threshold *d*. It is defined as:

$$R\left(d\right)=\frac{100}{|G|}\sum_{g\in G}‍I\left(\underset{r\in R}{\text{min}}∥\;g-r\;∥<d\right).$$ (4)

Its value ranges from 0 to 100, with higher values indicating better performance. Both the above 2 metrics are extensively utilized in recent studies [43,48].

3. F-score. The F-score, denoted as *F*(*d*), serves as a harmonic summary measure that encapsulates both the precision *P*(*d*) and recall *R*(*d*) for a given distance threshold *d*. It is specifically designed to penalize extreme imbalances between *P*(*d*) and *R*(*d*). Mathematically, it can be expressed as:

$$F\left(d\right)=\frac{2\times P\left(d\right)\times R\left(d\right)}{P\left(d\right)+R\left(d\right)}.$$ (5)

The harmonic nature of the F-score ensures that if either *P*(*d*) or *R*(*d*) approaches zero, the F-score will also tend toward zero, providing a more robust summary statistic than the arithmetic mean. F-score ranges from 0 to 100, with higher values indicating better performance. The details about value of *d* cutoff is given later in discussion about precision–recall curves.

For quantifying the quality of the NeRF-rendered 2D image compared to the validation image (left out from NeRF training), the following metrics are used:

4. LPIPS [49]: To quantify the perceptual differences between 2 image patches, *x* and *x*_0_, the LPIPS framework employs activations from a neural network *F*. Features are extracted from *L* layers and normalized across the channel dimension. For each layer *l*, the normalized features are represented by ${\hat{y}}^{l}$ and ${\hat{y}}^{l0}$, which exist in the space *ℝ*^*H_l_* × *W_l_* × *C_l_*^. These are then weighted channel-wise by a vector *w_l_* ∈ *ℝ^C_l_^*. The perceptual distance is computed using the *ℓ*_2_ norm, both spatially and across channels, as expressed in the equation:

$$d\left(x,x_{0}\right)=\sum_{l}‍\frac{1}{H_{l}W_{l}}\sum_{h,w}‍{\left\|w_{l}⊙\left({\hat{y}}_{\mathrm{hw}}^{l}-{\hat{y}}_{\mathrm{hw}}^{l0}\right)\right\|}_{2}^{2}$$ (6)

This distance metric, *d*(*x*, *x*_0_), provides a scalar value indicating the perceptual dissimilarity between the patches. The vector *w_l_* weights the contribution of each channel to the distance metric. By setting *w_l_* to $1/\sqrt{C_{l}}$, the computation effectively measures the cosine distance, highlighting the directional alignment of the feature vectors instead of their magnitude. Its value ranges from 0 to 1, with lower values indicating better performance.

5. Peak signal-to-noise ratio (PSNR) [50]: The PSNR between 2 images, one being the reference and the other the reconstructed image, is defined as:

$$\text{PSNR}=10\cdot {\text{log}}_{10}\left(\frac{{\mathrm{MAX}}_{I}^{2}}{\mathrm{MSE}}\right),$$ (7)

where MAX*_I_* is the maximum possible pixel value of the image, and MSE is the mean squared error between the reference and the reconstructed image. The MSE is given by:

$$\mathrm{MSE}=\frac{1}{\mathrm{mn}}\sum_{i=1}^{m}‍\sum_{j=1}^{n}‍{\left(I\left(i,j\right)-K\left(i,j\right)\right)}^{2},$$ (8)

where *I* is the reference image, *K* is the reconstructed image, and *m* and *n* are the dimensions of the images. A higher value of PSNR indicate better performance.

6. Structural Similarity Index (SSIM) [51]: The SSIM index is a method for predicting the perceived quality of digital television and cinematic pictures, as well as other kinds of digital images and videos. SSIM is designed to improve on traditional methods like PSNR and MSE, which have proven to be inconsistent with human eye perception. The SSIM index between 2 images *x* and *y* is defined as:

$$\text{SSIM}\left(x,y\right)=\frac{\left(2\mu_{x}\mu_{y}+C_{1}\right)\left(2\sigma_{\mathrm{xy}}+C_{2}\right)}{\left(\mu_{x}^{2}+\mu_{y}^{2}+C_{1}\right)\left(\sigma_{x}^{2}+\sigma_{y}^{2}+C_{2}\right)},$$ (9)

where *μ_x_* is the average of *x*, *μ_y_* is the average of *y*, $\sigma_{x}^{2}$ is the variance of *x*, $\sigma_{y}^{2}$ is the variance of *y*, *σ_xy_* is the covariance of *x* and *y*, and *C*_1_ and *C*_2_ are constants to stabilize the division with weak denominator. These last 3 metrics do not need the 3D ground truth and are widely used in literature [52,53] for evaluation. SSIM ranges from −1 to 1, with higher values indicating better performance.

Precision–Recall curves: Precision–recall curves are utilized to methodically evaluate how distance threshold *d* changes influence precision *P*(*d*) and recall *R*(*d*) metrics, demonstrating the trade-off between these measurements under varying threshold conditions. To set the value of *d* for the final assessment, we opt for a conservative estimate before the plateauing of precision–recall curves. For indoor scenarios, assuming a hypothetical grid size of 128 × 128 × 128 for reference, we establish *d* at 0.005. In this scenario, the voxel size is calculated as 1/128 ≈ 0.0078125, which makes the threshold of 0.005 smaller than the voxel size. This indicates a requirement for points to be closer than the dimensions of a single voxel to be identified as distinct, highlighting a prioritization of detail sensitivity within a hypothetically coarser grid. Such a setting is especially pertinent for capturing the complex geometries of indoor plants, where precision in detail is crucial. Due to the size and complexity of the scene, a threshold of 0.01 is selected for outdoor plant reconstructions.

### Early stopping of NeRF training using LPIPS

In training NeRFs for plant scene reconstruction, the F1 score is essential for validating the accuracy of the reconstructed point cloud against the ground truth. The inherent challenge during the training phase of NeRFs is the absence of ground truth, paradoxically the output we aim to correspond. Moreover, the training process for NeRFs is notoriously compute-intensive. The cumulative costs become challenging when scaled to multiple scenes or across extensive agricultural fields.

**Fig. 4.**
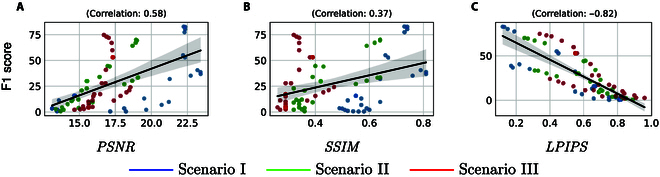
Correlation analysis between different metrics with F1 Score via Pearson coefficients: (A) PSNR, (B) SSIM, and (C) LPIPS.

Figure 4 shows the scatter plots of PSNR, SSIM, and LPIPS scores against the F1 score, alongside their respective Pearson correlation coefficients. This visualization offers an immediate visual assessment of the relationships between these metrics and allows for a nuanced understanding of how accurately each metric predicts the true F1 score. The exceptionally strong negative correlation between LPIPS and F1 score (−0.82) reinforces the notion that LPIPS effectively captures the perceptual similarity between the reconstructed and ground truth point clouds, making it a reliable proxy for F1 score, the ultimate measure of reconstruction fidelity.

The significant negative correlation between LPIPS and the F1 score (−0.82), PSNR (−0.81), and SSIM (−0.69) underscore the impact of LPIPS on the quality of 3D reconstruction (see the Supplementary Materials for detailed correlation matrix). The high magnitude of these coefficients, particularly the −0.82 with the F1 score, indicates that LPIPS is a robust predictor of reconstruction accuracy: as the perceptual similarity measure improves (meaning LPIPS decreases), the fidelity of the reconstructed point cloud to the ground truth improves correspondingly. This observation not only suggests the utility of LPIPS as a stand-in metric when the ground truth is unavailable but also highlights its potential as a more influential factor than traditional metrics such as PSNR (0.58) and SSIM (0.37) in determining the overall quality of NeRF-generated reconstructions.

Given this strong correlation, LPIPS emerges as a promising surrogate metric for early stopping during NeRF training. By monitoring LPIPS, one can infer the likely F1 score and make informed decisions about halting the training process. This method could decrease computational costs and time, as one need not await the completion of full training to predict its efficacy in terms of F1 score.


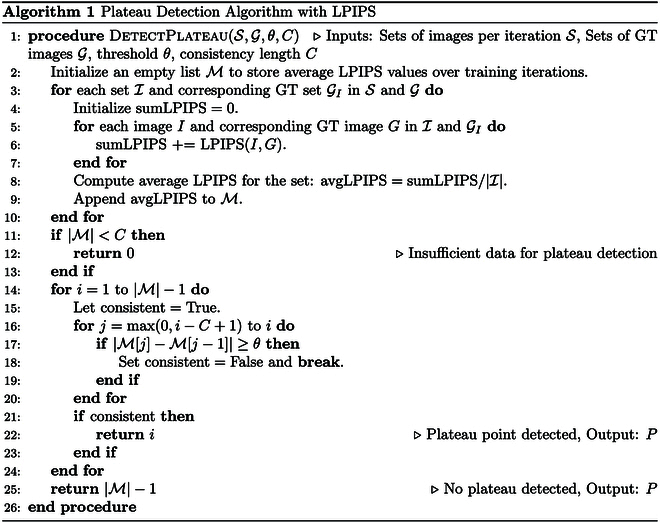


Algorithm for Plateau Detection: The plateau detection algorithm identifies a stabilization point in a series of metric values, such as LPIPS. The updated algorithm computes the average LPIPS for each set of images in S against their corresponding ground truth images in G. It then assesses the sequence of these average LPIPS values to identify a plateau, using a specified threshold *θ* and a consistency length *C*. The detection of the plateau point *P* is crucial for indicating an optimal stopping point in the training process. To validate the efficacy of the early stopping algorithm, we applied it to a diverse dataset comprising 5 plant types captured in both indoor and outdoor settings. The threshold (*θ*) was set to 0.005, and the consistency length (C) was fixed at 6. The granularity of interpolation was set to 1,000, spanning a total of 60,000 training iterations. These hyperparameters were chosen based on empirical observations to ensure a balance between computational efficiency and reconstruction accuracy.

## Results

**Fig. 5.**
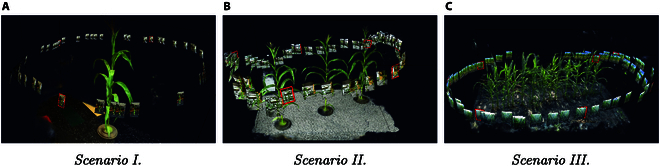
Camera pose estimations across 3 different scenarios. (A) Scenario I. (B) Scenario II. (C) Scenario III.

**Fig. 6.**
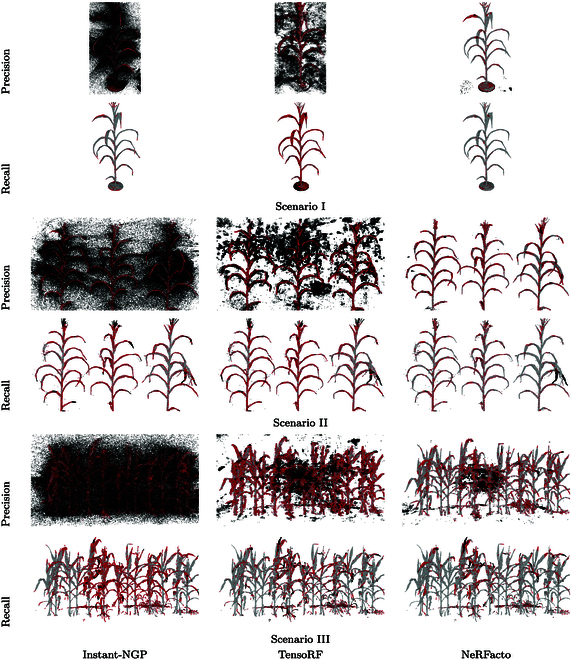
Precision and recall of 3D reconstruction using different NeRF techniques across different scenarios. Legend: 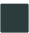 Correct, 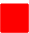 Missing, 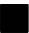 Outlier.

**Fig. 7.**
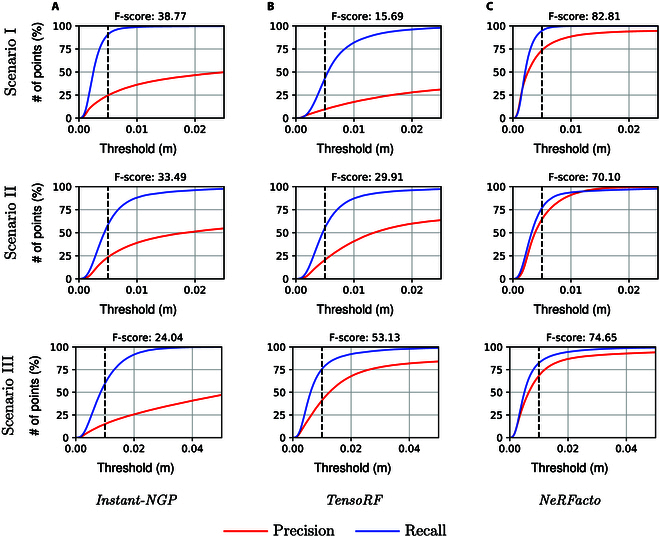
Precision-recall curves for the three scenarios based on varying distance thresholds after 30,000 iterations (Scenarios I and II) and 60,000 iterations (Scenario III) for (A) Instant-NGP, (B) TensoRF, and (C) NeRFacto.

We evaluated the performance of NeRF models across various scenarios, from controlled indoor environments to complex outdoor field conditions, using key performance metrics to assess their efficacy in 3D plant reconstruction. The NeRFs were trained on an NVIDIA A100 graphics processing unit (GPU) with 80GB GPU RAM attached to an AMD EPYC 7543 32-core central processing unit (CPU) with 503GB CPU RAM. Posttraining, the models are converted into point clouds with approximately a million points each. Estimated camera poses from COLMAP are visualized in Fig. 5, and a summary of the performance metrics of each of the 3 scenarios is given in Table 2. 3D evaluation metrics are presented in this section; for a more granular analysis of 2D image metrics, please refer to the Supplementary Materials. Visually, the performance of each model could be assessed using Precision and Recall as shown in Fig. 6. The Precision–Recall curves of the different scenarios for different threshold values are shown in Fig. 7.

**Table 2.** Performance metrics of NeRFs reconstruction techniques across Scenarios I, II, and III

#	Model	Precision↑	Recall↑	F1↑	PSNR↑	SSIM↑	LPIPS↓	Time (s)↓
I	Instant-NGP	24.66	90.62	38.77	23.41	0.81	0.17	756
	TensoRF	9.58	43.34	15.69	14.69	0.55	0.66	1,973
	NeRFacto	73.57	94.72	82.81	22.24	0.73	0.12	1,938
II	Instant-NGP	23.45	58.57	33.49	19.08	0.64	0.31	1,886
	TensoRF	20.5	55.34	29.91	15.54	0.42	0.56	2,607
	TensoRF	64.47	76.8	70.1	18.93	0.64	0.25	1,226
III	Instant-NGP	15.06	59.55	24.04	18.54	0.47	0.4	1,466
	TensoRF	40.95	75.62	53.13	17.32	0.39	0.55	1,965
	TensoRF	68.29	82.32	74.65	16.7	0.32	0.34	1,499

Visualization Color Code: The color-coded visualizations employed provide an intuitive understanding of spatial relationships within the 3D reconstructed plant structures. The interpretation of colors is as follows:

• Gray: (Correct) Represents points within a predefined distance threshold relative to the reference point cloud. This color indicates accurate points in precision and recall evaluations, where precision assesses the reconstruction against the ground truth, and recall evaluates the ground truth against the reconstruction.

• Red: (Missing) Depicts points in the point cloud being tested that are beyond the distance threshold but within 3 standard deviations from the nearest point in the reference point cloud. These points are considered inaccuracies, showing missing details in the reconstruction when assessing precision and highlighting missing elements in the ground truth during recall analysis.

• Black: (Outlier) Highlights points in the point cloud being tested that are more than 3 standard deviations away from any point in the reference point cloud. These points are extreme outliers and represent notable errors in the reconstruction relative to the ground truth for precision evaluations and similarly notable discrepancies in the ground truth relative to the reconstruction for recall.

### Scenario I - single plants indoors

We first look at the results of reconstructing a single plant in an indoor environment. Detailed evolution of each metric over training iterations is given in the Supplementary Materials.

Precision: For Scenario I, NeRFacto, achieved the highest precision followed by TensoRF and Instant-NGP (see Fig. 6) after 30,000 iterations. Across all models, precision generally increases with the number of iterations. 

Recall: The recall metric follows a similar trend, with Instant-NGP and NeRFacto showing increases with more iterations, indicating an enhanced ability to encompass points from the ground truth. Notably, NeRFacto achieves remarkably high recall values (over 90) at higher iterations, suggesting its superiority in the completeness of reconstruction. TensoRF’s recall values are markedly lower, indicating that it may miss more details from the ground truth compared to the other models.

F1 Score: The F1 score, balancing precision and recall, highlights NeRFacto as the most balanced model, especially at higher iterations, with scores above 80. Instant-NGP shows a substantial improvement in F1 scores as iterations increase, but it does not reach the same peak as NeRFacto. TensoRF lags in this metric, indicating a less balanced performance between precision and recall.

Computation Time: Time efficiency is a crucial factor, especially for practical applications. Instant-NGP demonstrates a relatively balanced approach between efficiency and performance, with time increments correlating reasonably with the increase in iterations. However, it becomes time-consuming at high iterations (20,000 and 30,000). NeRFacto, while showing better performance in many metrics, demands considerably more time, especially at higher iterations, which could be a limiting factor in time-sensitive scenarios. The evolution of precision over training time for NeRFacto is given in the Supplementary Materials. TensoRF, despite its lower performance in other metrics, maintains a more consistent time efficiency, suggesting its suitability for applications where time is a critical constraint. 

Overall Performance and Suitability: In sum, NeRFacto emerges as the most robust model in terms of precision, recall, F1 score, and image quality metrics (PSNR, SSIM, and LPIPS), making it highly suitable for applications demanding high accuracy and completeness in 3D modeling. However, its time inefficiency at higher iterations might restrict its use in time-sensitive contexts. Instant-NGP presents a good balance between performance and efficiency, making it a viable option for moderately demanding scenarios. Detailed results are given in Table 2, after complete training. The Precision–Recall curves based on varying distance threshold after maximum training of 30,000 iterations is given in Fig. 7.

Insight 1: Computational Cost and Accuracy Trade-off in Instant-NGP and NeRFacto: The steep increase in performance metrics with the number of iterations for both Instant-NGP and NeRFacto suggests that these models require a substantial amount of data processing to achieve high accuracy, which is critical in high-fidelity 3D modeling. However, this also implies a higher computational cost, which needs to be considered in practical applications.

Insight 2: Model Suitability in High-Detail 3D Reconstructions: The notable disparity in the performance of TensoRF compared to the other 2 models, particularly in precision and recall, indicates that not all NeRF models are equally suited for tasks requiring high-detail 3D reconstructions. This highlights the importance of model selection based on the specific requirements of the application.

Insight 3: Divergence in 2D Image Quality and 3D Reconstruction in Instant-NGP: A detailed examination reveals that Instant-NGP demonstrates strength in 2D image quality metrics such as PSNR, SSIM, and LPIPS, reflecting its ability to produce better rendered image quality. However, this excellence in 2D imaging does not correspondingly extend to 3D reconstruction metrics like Precision, Recall, and F1 Score. This observation highlights a notable distinction in the challenges associated with optimizing for high-quality image rendering as opposed to achieving accurate 3D representations. The model’s adeptness at rendering highly detailed 2D images does not necessarily imply its effectiveness in accurately reconstructing complex 3D structures, particularly in the context of intricate plant models. This insight underscores the need for a nuanced approach in evaluating the performance of models that are tasked with both 2D image rendering and 3D spatial reconstruction.

### Scenario II - multiple plants indoors

We observe marked differences in model behaviors compared to the single plant scenario, likely attributed to the added intricacy of multiple plants in a single scene. Detailed evolution of each metric over training iterations is given in the Supplementary Materials.

Precision: As shown in Fig. 6, Instant-NGP exhibits a steady increase in precision with more iterations, peaking at a high value. However, NeRFacto starts at a higher precision and reaches an even higher peak, indicating a more accurate reconstruction of the corn plants. TensoRF, although improving with more iterations, lags behind the others in terms of precision.

Recall: A similar pattern is observed for recall, with NeRFacto consistently maintaining a higher recall compared to the other methods, suggesting its ability to better encompass points in the ground truth. Both Instant-NGP and TensoRF exhibit increasing recall with more iterations but at lower levels than NeRFacto.

F1 Score: The F1 Score, balancing precision and recall, follows a similar trend. NeRFacto demonstrates the best balance between precision and recall, with its F1 score peaking at 70.10, while Instant-NGP and TensoRF achieve lower peak F1 scores.

Computation Time: The time taken for iterations is crucial for efficiency. Instant-NGP and NeRFacto have comparable times, but TensoRF takes substantially longer at higher iterations, indicating less time efficiency. 

Overall Performance and Suitability: NeRFacto emerges as the most balanced and efficient model, exhibiting high precision, recall, and F1 scores, along with favorable PSNR, SSIM, and LPIPS values. Its efficiency in time taken is also comparable to Instant-NGP. Instant-NGP, while showing improvements, does not quite match NeRFacto’s balance of precision and recall. TensoRF, despite its merits, falls behind in several key metrics, particularly in precision, recall, SSIM, and LPIPS. The results after complete training are given in Table 2. The Precision–Recall curves based on varying distance thresholds after maximum training of 30,000 iterations are given in Fig. 7.

Insight 1: Improved Performance of TensoRF in Scenario II: In the second scenario, TensoRF demonstrated an improvement compared to its performance in the first scenario. Specifically, its F1 score, a critical metric for 3D modeling accuracy, increased from 15.69 in the first scenario to 29.91 after 30,000 iterations in the second scenario. This improvement highlights TensoRF’s potential in more complex or demanding 3D modeling tasks, especially when allowed to complete its training process.

Insight 2: 2D Metrics Versus 3D F1 Score for Instant-NGP and NeRFacto: While Instant-NGP and NeRFacto show comparable results in 2D image quality metrics such as PSNR and SSIM, a distinct difference is observed in their 3D modeling capabilities, as reflected in their F1 scores, as observed in last scenario. This suggests that NeRFacto might be a more reliable choice for applications requiring high accuracy in 3D reconstructions.

### Scenario III - multiple plants outdoors

Scenario III is the most complex, with multiple overlapping plants captured in field conditions. The models were also trained until 60,000 iterations, while the previous 2 scenarios were trained only for 30,000 iterations. Detailed evolution of each metric over training iterations is given in the Supplementary Materials.

Precision: As observed in Fig. 6, NeRFacto consistently demonstrates the highest precision across all iterations, peaking at 68.29%, suggesting its ability to reconstruct points close to the ground truth. Instant-NGP shows a steady increase in precision with more iterations, while TensoRF, although starting lower, reaches a comparable precision to Instant-NGP at higher iterations.

Recall: NeRFacto leads in recall, achieving a high of 82.32%, indicating its effectiveness in encompassing points from the ground truth. Instant-NGP shows substantial improvement in recall with increased iterations but remains behind NeRFacto. TensoRF’s recall growth positions it between Instant-NGP and NeRFacto in terms of completeness.

F1 Score: Reflecting the balance between precision and recall, NeRFacto emerges as the superior model, with its F1 score peaking at 74.65%. Instant-NGP’s F1 score improves with more iterations but remains much lower, while TensoRF’s F1 score surpasses Instant-NGP, reaching 53.13%.

Computation Time: In terms of efficiency, Instant-NGP and NeRFacto are the fastest, followed by TensoRF.

Overall Performance and Suitability: NeRFacto again emerges as the most balanced and robust model, excelling in precision, recall, F1 score, and LPIPS. Detailed results are given in Table 2, after complete training. The Precision–Recall curves based on varying distance threshold after maximum training of 60,000 iterations is given in Fig. 7. The GPU memory usage of this scenario comes out to be approximately a constant 3GB (for the total memory of GPU being 80GB).

Insight 1: Enhanced Performance of TensoRF in Outdoor Settings: TensoRF demonstrates substantial improvement in its performance in the third scenario compared to the first. Specifically, its F1 score has seen a good increase, from 15.69 in the first scenario to 29.91 in the second and reaching 53.13 after 30,000 iterations in the current outdoor scenario. This upward trajectory in F1 scores, which is a balanced measure of precision and recall, indicates TensoRF’s enhanced capability in outdoor environments, potentially outperforming Instant-NGP in these settings. This suggests that TensoRF might be a more suitable choice for outdoor 3D modeling tasks where both precision and completeness are crucial. This property may have contributed in the selection of TensoRF as a building block for using multiple local radiance fields, during in-the-wild reconstruction [54].

Insight 2: LPIPS as a Strong Indicator of 3D Model Quality: The LPIPS metric appears to be a more representative measure of the quality of the resulting 3D models. In the analysis, we observe that models with lower LPIPS scores consistently show better performance across other metrics. This trend indicates the relevance of LPIPS in assessing the perceptual quality of 3D models. The further investigation into how LPIPS correlates with other metrics could provide deeper insights into model performance, especially in the context of realistic and perceptually accurate 3D reconstructions.

### Early stopping algorithm

The implementation of early stopping based on the LP-IPS metric yielded substantial savings in computational time across all scenarios, with a minor sacrifice in the fidelity of 3D reconstructions, as measured by the F1 score. Time savings were notable across the 3 tested methodologies—Instant-NGP, TensorRF, and NeRFacto—with each showing a marked decrease in training time without a commensurate loss in F1 score accuracy. For a deeper look of LPIPS, F1 Score and the recommended stopping point for each case, please consult the Supplementary Materials.

On average, the early stopping strategy resulted in a 61.1% reduction in training time, suggesting a substantial efficiency gain in the process of 3D plant reconstruction using NeRFs. Concurrently, the average F1 score loss was contained to 7.4%, indicating that the early plateau detection has a moderate impact on the quality of the 3D point cloud reconstructions. Specifically, Instant-NGP presented a more pronounced variation in F1 score loss, which was notably higher in Scenario III, thereby affecting its average loss more than TensorRF and NeRFacto. TensorRF and NeRFacto showed a remarkable consistency in time savings, which was mirrored in their comparable F1 score losses, highlighting the robustness of these methods in early stopping scenarios.

These findings articulate a compelling case for the utilization of early stopping in NeRF-based 3D reconstruction tasks, emphasizing the need to balance between computational resources and reconstruction precision. Such a balance is pivotal in scenarios where time efficiency is paramount yet a minimal compromise on reconstruction accuracy is permissible.

### Scenario IV - validation examples in field conditions

**Fig. 8.**
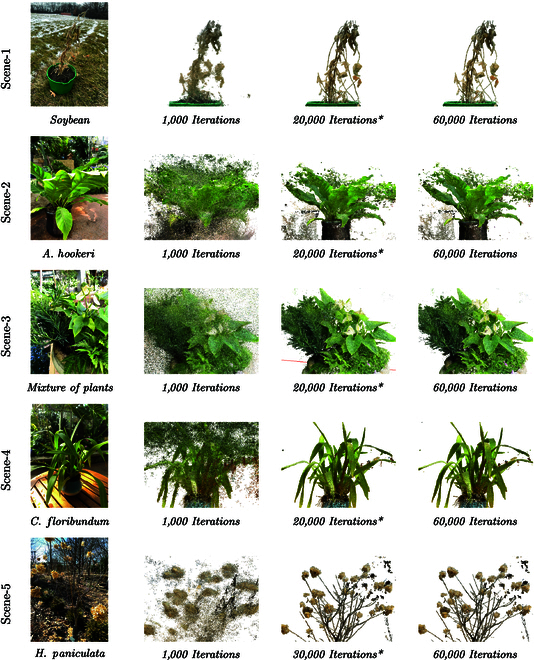
Scenes for validating the early stopping algorithm and their 3D reconstructions: original scenes in the first column, iterative reconstructions in the right column, and optimal iterations in the third column (*).

The efficacy of the LPIPS-based early stopping algorithm was validated using a diverse dataset comprising images from 5 different types of plants captured in both indoor and outdoor settings, as illustrated in Fig. 8. The validation process employed a threshold *θ* set to 0.005 and a consistency length *C* of 6, with the granularity of interpolation fixed at 1,000, spanning a total of 60,000 training iterations. For practical application, checkpoints, inherently exponential in nature, necessitated linear interpolation to facilitate algorithm execution. Figure 8 shows the rendered point clouds at 3 stages: after 1,000 iterations, at the recommended early stopping iteration, and upon completing the full 60,000 iterations of training. Each row of the figure corresponds to one of the 5 validation scenes, providing a qualitative comparative analysis.

Notably, for all indoor scenes, the algorithm recommended halting training at 20,000 iterations, whereas for outdoor scenes, the suggestion extended to 30,000 iterations. This distinction underscores the algorithm’s sensitivity to environmental variables affecting perceptual similarity metrics. The rendered point clouds, particularly at the early stopping points, exhibit minimal visual discrepancies when compared to those obtained after the full training duration. By reducing computational demands without much loss in fidelity, this approach is a cost-effective strategy for enhancing modeling throughput in precision agriculture and botanical research. We substantiate the hypothesis that LPIPS can serve as a reliable surrogate for direct F1 score estimation in the context of NeRF training. The algorithm’s ability to accurately predict optimal stopping points—balancing computational efficiency with reconstruction accuracy—presents a compelling case for its adoption in scenarios where resource conservation is paramount, yet quality cannot be entirely sacrificed.

## Discussion

In this section, we discuss the findings from our comparative analysis of NeRF models for 3D plant reconstruction. The results indicate that the Nerfacto model achieved the best performance, and we explore the theoretical basis for its superiority by examining the sampling strategies employed by the different models. Understanding these strategies provides insights into why Nerfacto outperformed the other models in terms of reconstruction quality. In our experiments, we found that the Nerfacto model produced the highest quality 3D reconstructions compared to Instant-NGP and other NeRF models. To understand the theoretical basis for Nerfacto’s superior performance, in this section, we take a deeper look at the sampling strategies used by Nerfacto and Instant-NGP and how they influence the visual quality and level of detail in the rendered scenes.

The divergent performance of the NeRF models necessitates a deeper examination of their underlying sampling strategies and their influence on the quality of 3D reconstruction. The difference in the output quality between Instant-NGP and Nerfacto, especially concerning the density and crispness of the rendered scenes, could indeed be related to the sampling strategies used by each algorithm.

Instant-NGP Sampling Strategy: Instant-NGP uses an improved training and rendering algorithm that involves a ray marching scheme with an occupancy grid. This means that when the algorithm shoots rays into the scene to sample colors and densities, it uses an occupancy grid to skip over empty space, as well as areas behind high-density regions to improve efficiency.

• The occupancy grid used in Instant-NGP is a multiscale grid that coarsely marks empty and nonempty space and is used to determine where to skip samples to speed up processing.

• This approach is quite effective in terms of speed, leading to substantial improvements over naive sampling methods.

• However, if the occupancy grid is not fine-grained enough or if the method for updating this grid is not capturing the scene’s density variations accurately, it could lead to a “muddy” or overly dense rendering because it might not be sampling the necessary areas with enough precision.

NeRFacto Sampling Strategy: Nerfacto, on the other hand, uses a combination of different sampling techniques:

• Camera Pose Refinement*:* By refining camera poses, Nerfacto ensures that the samples taken are based on more accurate viewpoints, which directly affects the clarity of the rendered images.

• Piecewise Sampler: This sampler is used to produce an initial set of samples, with a distribution that allows both dense sampling near the camera and appropriate sampling further away. This could lead to clearer images since it captures details both near and far from the camera.

• Proposal Sampler: This is a key part of the Nerfacto method. It uses a proposal network to concentrate sample locations in regions that contribute most to the final render, usually around the first surface intersection. This targeted sampling could be a major reason why Nerfacto produces crisper images—it focuses computational resources on the most visually relevant parts of the scene.

• Density Field: By using a density field guided by a hash encoding and a small fused MLP, Nerfacto can efficiently guide sampling even further. It does not require an extremely detailed density map since it is used primarily for guiding the sampling process, which means that it balances quality and speed without necessarily impacting the final image’s detail.

Instant-NGP’s sampling strategy is built for speed, with an occupancy grid that helps skip irrelevant samples. This approach is great for real-time applications but can potentially miss subtle density variations, leading to a denser and less clear output if the grid is not capturing all the necessary detail. Nerfacto’s sampling strategy is more complex and layered, with multiple mechanisms in place to ensure that sampling is done more effectively in areas that greatly affect the visual output. The combination of pose refinement, piecewise sampling, proposal sampling, and an efficient density field leads to more accurate sampling, which in turn produces crisper images. In summary, the reason for Nerfacto’s better reconstruction likely stems from its more refined and targeted approach to sampling, which concentrates computational efforts on the most visually impactful parts of the scene. In contrast, Instant-NGP’s faster but less targeted sampling may result in less clarity and more visual artifacts.

Finally, to retrieve the scale of the 3D reconstruction in the absence of reference point cloud data, a known scale can be placed on the ground during data collection. The exported point cloud can then be proportionally scaled based on this reference scale, which allows the size of the reconstructed plant to be calibrated to match its real-world dimensions. In order to show the practicality of this approach, we placed a 3D printed sphere of known diameter for the plant in Scenario I and captured the images. We then go through our pipeline of NeRF reconstruction, and instead of registering and scaling the scene to the ground truth LiDAR data, we scale it to the known sphere size. We then measured the height of the plant in this scenario. We find that by using this approach, the error in the height of the plant is within 1%. We provide additional details of this experiment in the Supplementary Materials. We note that this is a preliminary result, and more detailed studies need to be performed in the future on extracting the correct scale from NeRF reconstructions.

In conclusion, the findings of this research underscore the value of NeRFs as a nondestructive approach for 3D plant reconstruction in precision agriculture. Our methodology more effectively facilitates critical agricultural tasks, such as growth monitoring, yield prediction, and early disease detection from accurate reconstruction of plant structures. Our comparative analysis, which benchmarks different NeRF models against ground truth data, highlights the method’s efficiency, achieving a 74.65% F1 score within 30 min of GPU training. Introducing an early stopping algorithm based on LPIPS further enhances this process, reducing training time by 61.1% while limiting the average F1 score loss to just 7.4%.

Additionally, our work provides a comprehensive dataset and an evaluation framework, aiding the validation of current models and serving as a foundation for developing future NeRF applications in agriculture. The detailed insights into model performance across varied scenarios, coupled with the early stopping case study, offer practical guidance for 3D reconstruction using NeRFs. This research supports the advancement of nonintrusive agricultural technologies and also sets a baseline for future work at the intersection of NeRF technologies and agriculture, aiming to improve efficiency and accuracy in plant phenotyping and breeding.

## Data Availability

The data for the 4 scenarios including raw images and the point cloud will be made available online. The GIT repository containing the different implementations of the NeRF code will also be made public.
