# Supplementary material for: Evaluating Neural Radiance Fields for 3D Plant Geometry Reconstruction in Field Conditions
Source: Plant Phenomics. 2024 Sep 9;6:0235. doi: 10.34133/plantphenomics.0235 (PMC11382571; doi:10.34133/plantphenomics.0235)
Supplement: Supplementary 1 — Figs. S1 to S9 Tables S1 to S5 [file plantphenomics.0235.f1.zip › Supplementary Materials.pdf]

# Supplementary Material

The Supplement provides additional information on interpreting the visualizations and detailed metrics at different points of the training process for all scenarios. We also detail additional visualizations to support the validity of the proposed LPIPS-based early-stopping algorithms.

# S1 Visualization of 3D Reconstruction Metrics

The interpretation of the colors in the precision and recall figures (see Figure S1) is as follows:

- **Grey**: (Correct) Represents points within a predefined distance threshold relative to the reference point cloud. This color indicates accurate points in precision and recall evaluations, where precision assesses the reconstruction against the ground truth, and recall evaluates the ground truth against the reconstruction.
- **Red**: (Missing) Depicts points in the point cloud being tested that are beyond the distance threshold but within 3 standard deviations from the nearest point in the reference point cloud. These points are considered inaccuracies, showing missing details in the reconstruction when assessing precision and highlighting missing elements in the ground truth during recall analysis.
- **Black**: (Outlier) Highlights points in the point cloud being tested that are more than 3 standard deviations away from any point in the reference point cloud. These points are extreme outliers and represent significant errors in the reconstruction relative to the ground truth for precision evaluations, and similarly significant discrepancies in the ground truth relative to the reconstruction for recall.

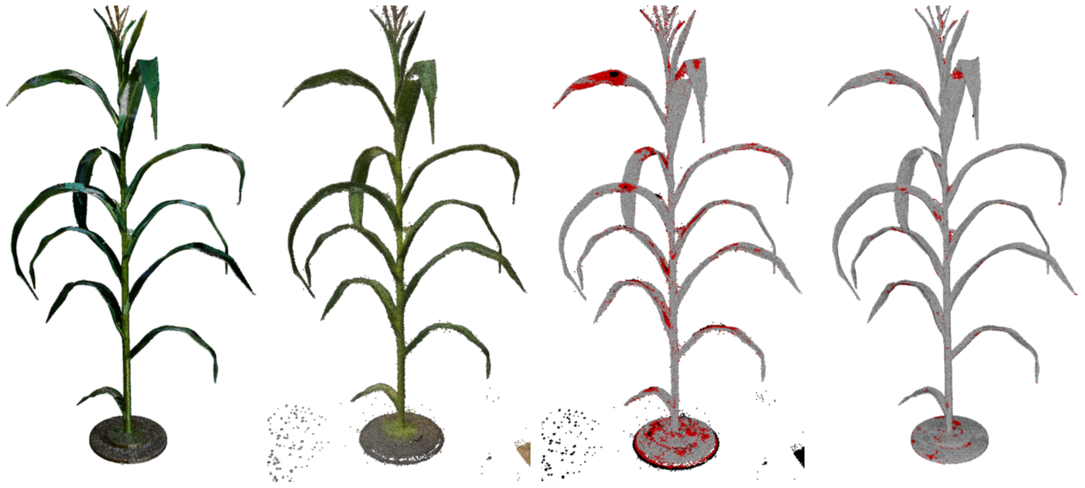

**(a)** *Ground Truth* **(b)** *Reconstruction* **(c)** *Precision* **(d)** *Recall*

**Figure S1:** *Point cloud 3D reconstruction metrics: (a) Original data; (b) Reconstruction; (c) Precision; (d) Recall. Legend:* ■ *indicates correct points,* ■ *indicates missing points, and* ■ *indicates outliers.*

# S2 Additional Performance Metric Results

Granular explanation of 2D metrics (PSNR, SSIM and LPIPS), and their evolution over the training process for each scenario is given below.

## S2.1 Scenario I

The trend of all metrics throughout the training process is depicted in Figure S4.
**Precision:** Instant-NGP shows a significant leap in precision from 100 to 5000 iterations (0.29 to 21.93), indicating a drastic improvement in the accuracy of reconstructed points relative to the ground truth. NeRFacto demonstrates a more consistent and steep rise in precision, reaching a peak of 73.57 at 30000 iterations, which surpasses Instant-NGP's best precision. TensoRF, however, shows a relatively modest increase in precision, suggesting its limited capability in accurately capturing fine details compared to the other two models. Visuals of precision at different points of the training is shown in Figure S2.
**PSNR:** The Peak Signal-to-Noise Ratio (PSNR) reflects the quality of rendered images. In this metric, Instant-NGP and NeRFacto show a gradual increase in PSNR with more iterations, suggesting improved image quality. TensoRF's PSNR values are lower, indicating potentially lower image quality throughout its iterations.
**SSIM:** The Structural Similarity Index (SSIM) is another measure of image quality, assessing the perceived change in structural information. Here, NeRFacto and Instant-NGP both show a steady increase in SSIM with more iterations, with NeRFacto achieving slightly higher scores, suggesting better preservation of structural information in its renderings. TensoRF, again, shows relatively lower SSIM scores.
**LPIPS:** The Lower Perceptual Image Patch Similarity (LPIPS) metric indicates perceived image similarity, with lower values being better. NeRFacto and Instant-NGP both show a significant decrease in LPIPS with more iterations, indicating improved perceptual similarity to the ground truth. TensoRF's LPIPS values are consistently higher, suggesting lower perceptual similarity.

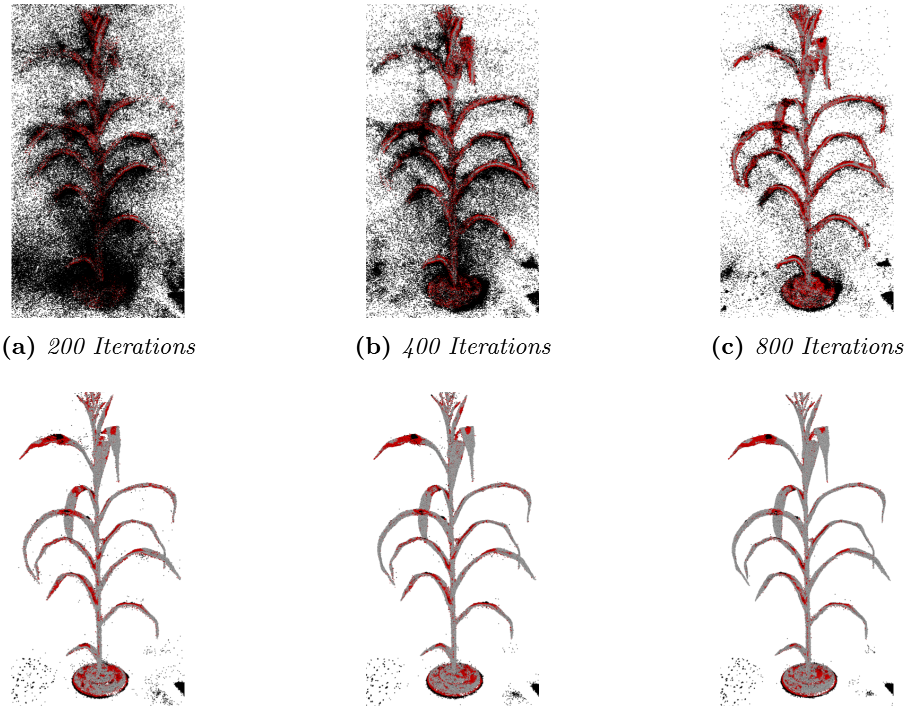

**(a)** *200 Iterations* **(b)** *400 Iterations* **(c)** *800 Iterations*

**(d)** *5000 Iterations* **(e)** *10000 Iterations* **(f)** *30000 Iterations*

**Figure S2:** *Precision change over iterations for NeRFacto - Scenario I. Legend:* ■ *Correct,* ■ *Missing,* ■ *Outlier.*

**Table S1:** *Detailed performance metrics of NeRFs reconstruction techniques - Scenario I*

| Model Name | Iters | Precision ↑ | Recall ↑ | F1 ↑ | PSNR ↑ | SSIM ↑ | LPIPS ↓ | T (s) ↓ |
|---|---|---|---|---|---|---|---|---|
| Instant-NGP | 100 | 0.29 | 2.48 | 0.53 | 17.14 | 0.58 | 0.81 | 13 |
| | 200 | 0.32 | 1.50 | 0.53 | 18.23 | 0.56 | 0.75 | 26 |
| | 400 | 1.25 | 7.14 | 2.13 | 19.64 | 0.60 | 0.66 | 42 |
| | 800 | 5.66 | 35.17 | 9.74 | 21.21 | 0.64 | 0.55 | 61 |
| | 1000 | 3.93 | 27.61 | 6.88 | 20.71 | 0.57 | 0.59 | 76 |
| | 5000 | 21.93 | 89.03 | 35.20 | 22.73 | 0.76 | 0.28 | 175 |
| | 10000 | 25.98 | 92.59 | 40.57 | 23.20 | 0.79 | 0.22 | 297 |
| | 20000 | 23.21 | 88.38 | 36.77 | 23.42 | 0.81 | 0.18 | 527 |
| | 30000 | 24.66 | 90.62 | 38.77 | 23.41 | 0.81 | 0.17 | 756 |
| TensoRF | 100 | 0.43 | 2.27 | 0.72 | 13.44 | 0.54 | 0.82 | 13 |
| | 200 | 0.65 | 4.47 | 1.13 | 13.55 | 0.52 | 0.82 | 25 |
| | 400 | 1.18 | 8.76 | 2.07 | 13.51 | 0.51 | 0.81 | 40 |
| | 800 | 1.86 | 14.22 | 3.29 | 13.10 | 0.50 | 0.79 | 61 |
| | 1000 | 2.05 | 16.40 | 3.65 | 13.14 | 0.49 | 0.79 | 77 |
| | 5000 | 6.63 | 36.57 | 11.22 | 13.59 | 0.52 | 0.70 | 420 |
| | 10000 | 9.58 | 43.47 | 15.69 | 14.64 | 0.55 | 0.67 | 859 |
| | 20000 | 9.51 | 43.19 | 15.59 | 14.68 | 0.55 | 0.67 | 1651 |
| | 30000 | 9.58 | 43.34 | 15.69 | 14.69 | 0.55 | 0.66 | 1973 |
| NeRFacto | 100 | 1.94 | 20.98 | 3.55 | 18.11 | 0.59 | 0.75 | 14 |
| | 200 | 7.72 | 42.84 | 13.08 | 19.50 | 0.57 | 0.65 | 27 |
| | 400 | 20.86 | 68.64 | 32.00 | 21.27 | 0.64 | 0.55 | 43 |
| | 800 | 39.48 | 80.26 | 52.92 | 22.33 | 0.67 | 0.45 | 64 |
| | 1000 | 41.35 | 82.54 | 55.09 | 22.20 | 0.66 | 0.44 | 79 |
| | 5000 | 66.43 | 92.51 | 77.33 | 22.30 | 0.73 | 0.19 | 430 |
| | 10000 | 70.04 | 93.94 | 80.25 | 22.34 | 0.74 | 0.15 | 564 |
| | 20000 | 73.32 | 94.51 | 82.58 | 22.35 | 0.74 | 0.13 | 1068 |
| | 30000 | 73.57 | 94.72 | 82.81 | 22.24 | 0.73 | 0.12 | 1938 |

## S2.2 Scenario II

Figure S3 illustrates the change of precision over the course of the training iterations. The trend of all metrics throughout the training process is depicted in Figure S5. For the same scenario, we show that Mip-NeRF fails to produce a reasonable reconstruction (Table S2).

**PSNR:** In terms of PSNR, which evaluates the quality of rendered images, all models show improvement with more iterations. Instant-NGP goes from 13.70 to 19.08, NeRFacto from 14.93 to 18.93, and TensoRF from 13.38 to 15.54. Instant-NGP achieves the highest PSNR, suggesting better image quality.

**SSIM:** For SSIM, higher values indicate better image structure similarity. Instant-NGP progresses from 0.36 to 0.64, NeRFacto from 0.35 to 0.64, and TensoRF from 0.35 to 0.42. Both Instant-NGP and NeRFacto perform similarly and better than TensoRF in this aspect.

**LPIPS:** Lower LPIPS values signify higher perceptual similarity to the ground truth. Instant-NGP decreases from 0.89 to 0.31, NeRFacto from 0.77 to 0.25, and TensoRF from 0.83 to 0.56, with NeRFacto showing the best perceptual image quality.

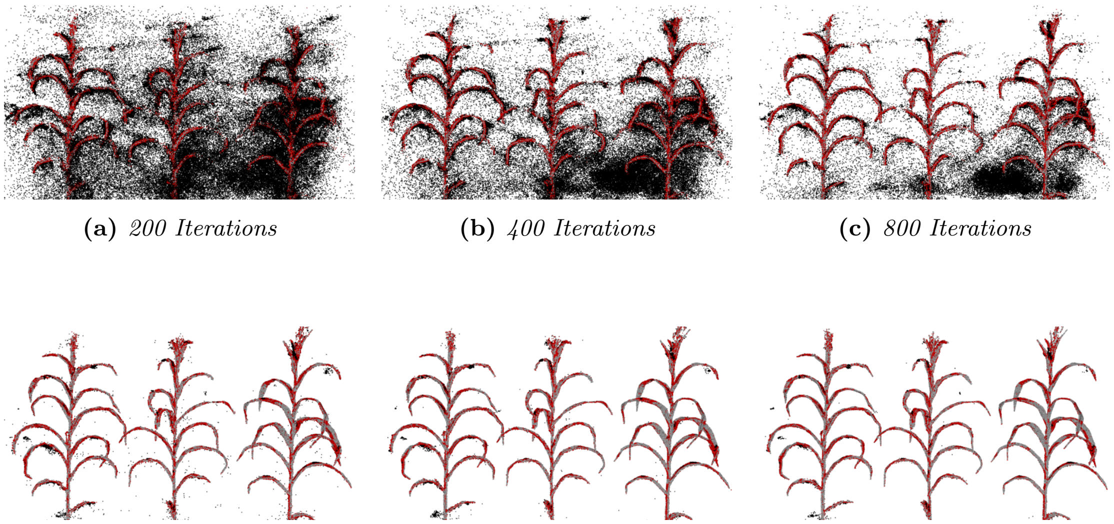

**(a)** *200 Iterations* **(b)** *400 Iterations* **(c)** *800 Iterations*

**(d)** *5000 Iterations* **(e)** *10000 Iterations* **(f)** *30000 Iterations*

**Figure S3:** *Precision change as a function of different training lengths for NeRFacto - Scenario II. Legend:* ■ *Correct,* ■ *Missing,* ■ *Outlier.*

**Table S2:** *Performance metrics of MipNeRF reconstruction - Scenario II (failed).*

| Iters | Precision ↑ | Recall ↑ | F1 ↑ | PSNR ↑ | SSIM ↑ | LPIPS ↓ | T (s) ↓ |
|---|---|---|---|---|---|---|---|
| 100 | 0.47 | 7.57 | 0.89 | 11.01 | 0.34 | 0.91 | 26 |
| 200 | 0.63 | 10.67 | 1.19 | 11.24 | 0.34 | 0.93 | 52 |
| 400 | 0.69 | 12.1 | 1.3 | 11.07 | 0.34 | 0.92 | 86 |
| 800 | 0.48 | 7.68 | 0.91 | 9.88 | 0.33 | 0.94 | 158 |
| 1000 | 0.44 | 7.19 | 0.82 | 9.65 | 0.32 | 0.94 | 192 |
| 5000 | 0.35 | 5.41 | 0.66 | 9.31 | 0.31 | 0.94 | 687 |
| 10000 | 0.42 | 6.29 | 0.79 | 8.88 | 0.29 | 0.9 | 1303 |
| 20000 | 0.34 | 5.3 | 0.65 | 8.78 | 0.28 | 0.89 | 2508 |
| 30000 | 0.35 | 5.71 | 0.67 | 9 | 0.27 | 0.88 | 3856 |

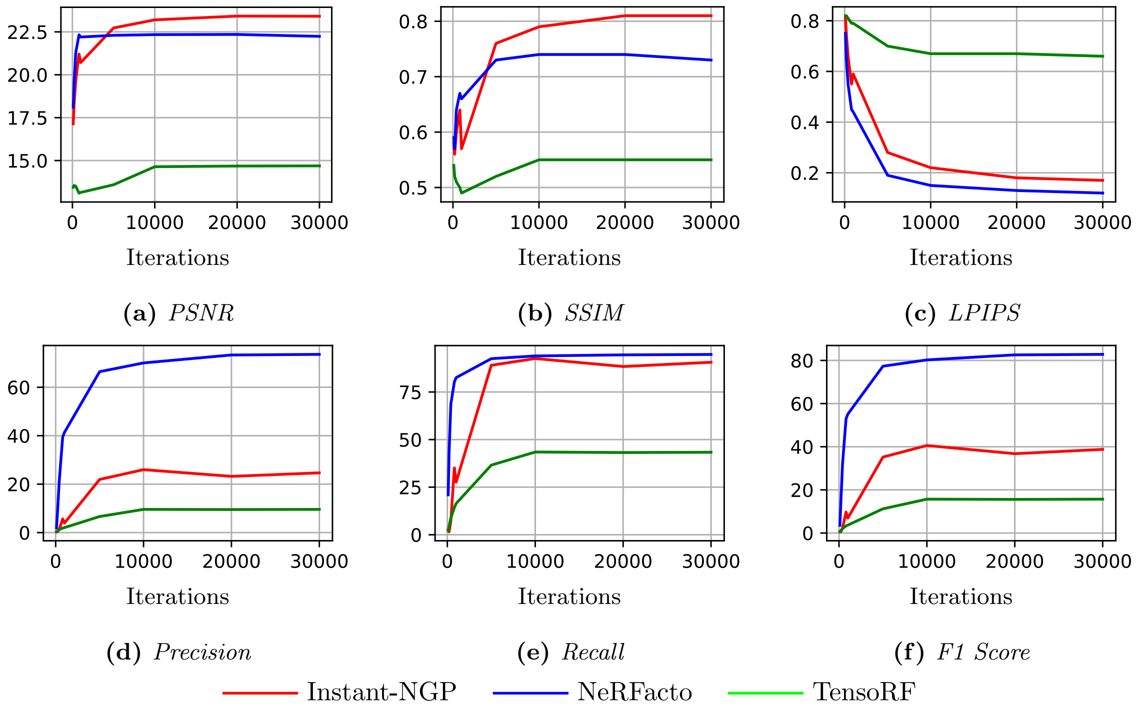


**Figure S4:** *Comparison of 2D quality (top) and 3D geometry (bottom) metrics for Scenario-I.*

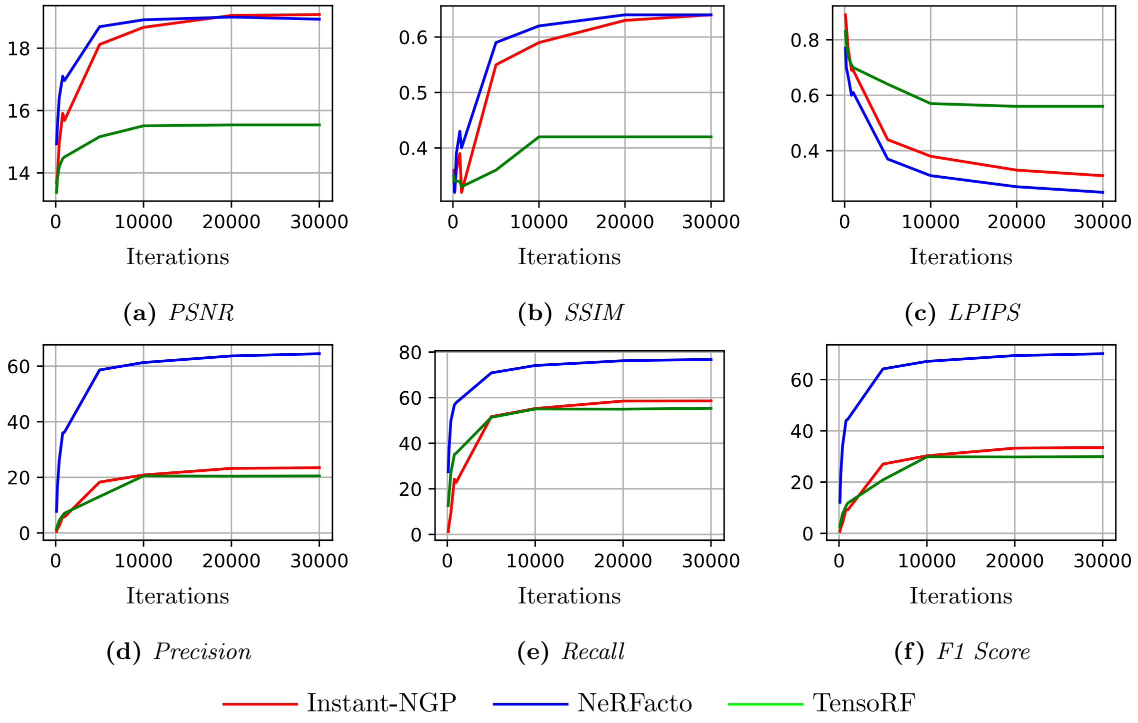


**Figure S5:** *Comparison of 2D quality (top) and 3D geometry (bottom) metrics for Scenario-II*

**Table S3:** *Detailed performance metrics of NeRFs reconstruction techniques - Scenario II*

| Model Name | Iters | Precision ↑ | Recall ↑ | F1 ↑ | PSNR ↑ | SSIM ↑ | LPIPS ↓ | T (s) ↓ |
|---|---|---|---|---|---|---|---|---|
| Instant-NGP | 100 | 0.57 | 1.23 | 0.78 | 13.70 | 0.36 | 0.89 | 13 |
| | 200 | 1.52 | 4.41 | 2.27 | 13.91 | 0.32 | 0.85 | 27 |
| | 400 | 2.49 | 9.35 | 3.93 | 14.95 | 0.36 | 0.77 | 43 |
| | 800 | 5.83 | 24.29 | 9.41 | 15.91 | 0.39 | 0.69 | 63 |
| | 1000 | 5.72 | 22.80 | 9.14 | 15.68 | 0.32 | 0.69 | 78 |
| | 5000 | 18.30 | 51.68 | 27.02 | 18.12 | 0.55 | 0.44 | 176 |
| | 10000 | 20.86 | 55.25 | 30.28 | 18.67 | 0.59 | 0.38 | 296 |
| | 20000 | 23.24 | 58.53 | 33.27 | 19.05 | 0.63 | 0.33 | 1023 |
| | 30000 | 23.45 | 58.57 | 33.49 | 19.08 | 0.64 | 0.31 | 1886 |
| TensoRF | 100 | 1.51 | 12.62 | 2.70 | 13.38 | 0.35 | 0.83 | 14 |
| | 200 | 2.53 | 17.53 | 4.43 | 13.81 | 0.34 | 0.79 | 27 |
| | 400 | 4.41 | 26.94 | 7.58 | 14.19 | 0.34 | 0.74 | 44 |
| | 800 | 6.28 | 35.10 | 10.66 | 14.46 | 0.34 | 0.71 | 66 |
| | 1000 | 7.09 | 35.61 | 11.82 | 14.51 | 0.33 | 0.70 | 82 |
| | 5000 | 13.09 | 51.34 | 20.86 | 15.16 | 0.36 | 0.64 | 399 |
| | 10000 | 20.51 | 55.03 | 29.88 | 15.51 | 0.42 | 0.57 | 840 |
| | 20000 | 20.44 | 54.97 | 29.80 | 15.54 | 0.42 | 0.56 | 1709 |
| | 30000 | 20.50 | 55.34 | 29.91 | 15.54 | 0.42 | 0.56 | 2607 |
| NeRFacto | 100 | 7.77 | 27.44 | 12.12 | 14.93 | 0.35 | 0.77 | 15 |
| | 200 | 16.42 | 37.77 | 22.89 | 15.51 | 0.32 | 0.70 | 28 |
| | 400 | 25.83 | 49.81 | 34.02 | 16.40 | 0.39 | 0.67 | 44 |
| | 800 | 35.97 | 56.86 | 44.07 | 17.10 | 0.43 | 0.60 | 64 |
| | 1000 | 36.22 | 57.80 | 44.53 | 16.96 | 0.40 | 0.61 | 79 |
| | 5000 | 58.64 | 70.87 | 64.18 | 18.69 | 0.59 | 0.37 | 404 |
| | 10000 | 61.31 | 74.12 | 67.11 | 18.91 | 0.62 | 0.31 | 749 |
| | 20000 | 63.68 | 76.21 | 69.38 | 19.00 | 0.64 | 0.27 | 988 |
| | 30000 | 64.47 | 76.80 | 70.10 | 18.93 | 0.64 | 0.25 | 1226 |

## S2.3 Scenario III

The trend of all metrics throughout the training process is depicted in Figure S6.

**PSNR:** In terms of image quality, as measured by PSNR, all models show incremental improvements with more iterations. Instant-NGP and NeRFacto display similar trends, with NeRFacto slightly leading, peaking at 16.70 at 60000 iterations. TensoRF shows a comparable maximum PSNR of 17.32, suggesting its slight edge in rendering higher-quality images.

**SSIM:** For the SSIM metric, all three models show improvements with increased iterations. NeRFacto maintains a slight advantage over the others, peaking at 0.32 at 60000 iterations, indicating its better performance in maintaining structural integrity in the rendered images. Instant-NGP and TensoRF show similar SSIM scores, with TensoRF slightly leading at higher iterations.

**LPIPS:** The LPIPS scores, which assess perceptual similarity, decrease for all models with more iterations, indicating improved performance. NeRFacto and TensoRF show similar trends, with NeRFacto having a slight edge, achieving a score of 0.34 at 60000 iterations compared to TensoRF's 0.55. Instant-NGP's performance is consistently lower in this metric.

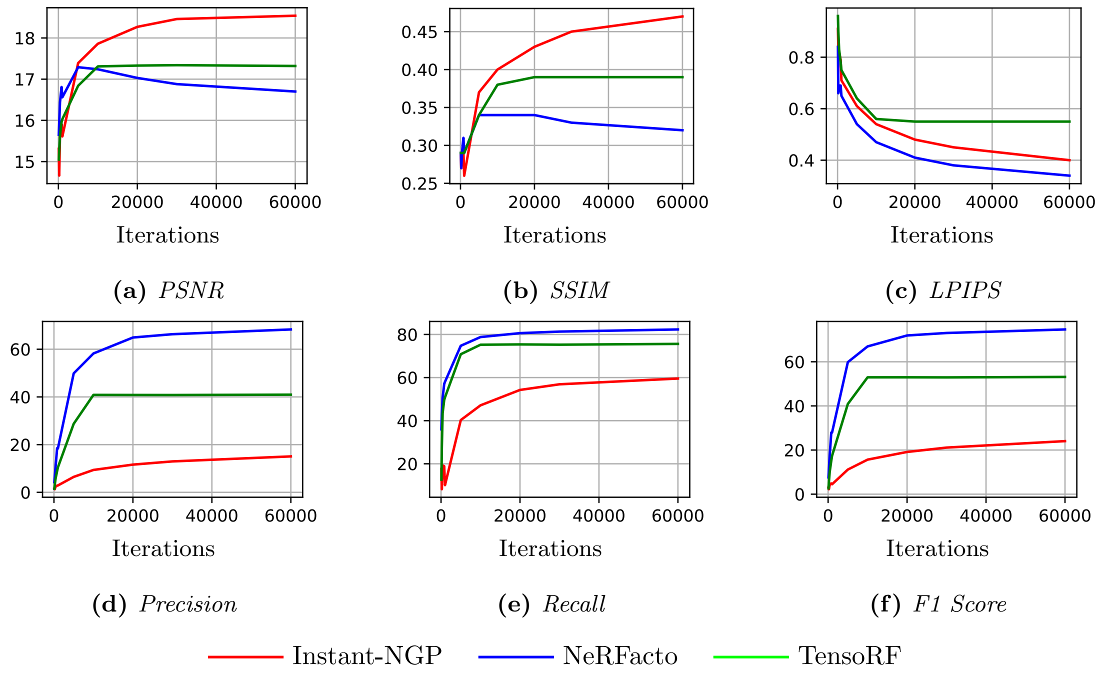


**(a)** *PSNR* **(b)** *SSIM* **(c)** *LPIPS*

**(d)** *Precision* **(e)** *Recall* **(f)** *F1 Score*

**Figure S6:** *Comparison of 2D quality (top) and 3D geometry (bottom) metrics for Scenario-III.*

Detailed metrics for all the three scenarios are given in the tables: Table S1, Table S3, and Table S4.

## S2.4 Validation of Early-Stopping Algorithm

Detailed look of LPIPS metric plots across various validation scenes alongside the algorithmically proposed early stopping points, which does not significantly compromise the reconstruction quality, is also provided. For a deeper look of LPIPS, F1 Score and the recommended stopping point for each case, consult Figure S7 and Figure S8. The different number of images used for the different scenarios is provided in Table S5.

**Table S4:** *Detailed performance metrics of NeRFs reconstruction techniques - Scenario III*

| Model Name | Iters | Precision ↑ | Recall ↑ | F1 ↑ | PSNR ↑ | SSIM ↑ | LPIPS ↓ | T (s) ↓ |
|---|---|---|---|---|---|---|---|---|
| Instant-NGP | 100 | 1.61 | 17.61 | 2.95 | 15.31 | 0.29 | 0.91 | 16 |
| | 200 | 1.29 | 8.19 | 2.23 | 14.66 | 0.27 | 0.84 | 30 |
| | 400 | 2.45 | 19.22 | 4.34 | 15.65 | 0.29 | 0.82 | 46 |
| | 800 | 2.81 | 18.90 | 4.89 | 15.91 | 0.29 | 0.79 | 67 |
| | 1000 | 2.88 | 10.08 | 4.48 | 15.61 | 0.26 | 0.71 | 82 |
| | 5000 | 6.47 | 40.28 | 11.15 | 17.39 | 0.37 | 0.61 | 186 |
| | 10000 | 9.38 | 47.12 | 15.65 | 17.86 | 0.40 | 0.54 | 311 |
| | 20000 | 11.62 | 54.26 | 19.13 | 18.27 | 0.43 | 0.48 | 548 |
| | 30000 | 12.96 | 56.88 | 21.11 | 18.46 | 0.45 | 0.45 | 783 |
| | 60000 | 15.06 | 59.55 | 24.04 | 18.54 | 0.47 | 0.40 | 1466 |
| TensoRF | 100 | 1.48 | 12.65 | 2.65 | 15.05 | 0.29 | 0.96 | 17 |
| | 200 | 3.05 | 27.40 | 5.48 | 15.26 | 0.29 | 0.90 | 31 |
| | 400 | 5.69 | 43.45 | 10.06 | 15.63 | 0.29 | 0.83 | 47 |
| | 800 | 8.92 | 49.59 | 15.13 | 15.96 | 0.29 | 0.78 | 68 |
| | 1000 | 10.48 | 50.82 | 17.37 | 16.03 | 0.29 | 0.75 | 84 |
| | 5000 | 28.74 | 70.83 | 40.89 | 16.84 | 0.34 | 0.64 | 208 |
| | 10000 | 40.85 | 75.24 | 52.95 | 17.31 | 0.38 | 0.56 | 374 |
| | 20000 | 40.82 | 75.38 | 52.96 | 17.33 | 0.39 | 0.55 | 697 |
| | 30000 | 40.80 | 75.26 | 52.92 | 17.34 | 0.39 | 0.55 | 1018 |
| | 60000 | 40.95 | 75.62 | 53.13 | 17.32 | 0.39 | 0.55 | 1965 |
| NeRFacto | 100 | 4.26 | 35.99 | 7.61 | 15.65 | 0.29 | 0.84 | 13 |
| | 200 | 6.53 | 42.49 | 11.31 | 15.91 | 0.27 | 0.66 | 27 |
| | 400 | 10.48 | 50.45 | 17.35 | 16.44 | 0.29 | 0.69 | 43 |
| | 800 | 18.49 | 57.01 | 27.93 | 16.81 | 0.31 | 0.69 | 64 |
| | 1000 | 18.39 | 58.02 | 27.93 | 16.56 | 0.29 | 0.65 | 80 |
| | 5000 | 49.87 | 74.75 | 59.83 | 17.29 | 0.34 | 0.54 | 189 |
| | 10000 | 58.22 | 78.80 | 66.96 | 17.24 | 0.34 | 0.47 | 318 |
| | 20000 | 64.91 | 80.61 | 71.92 | 17.03 | 0.34 | 0.41 | 561 |
| | 30000 | 66.30 | 81.33 | 73.05 | 16.88 | 0.33 | 0.38 | 803 |
| | 60000 | 68.29 | 82.32 | 74.65 | 16.70 | 0.32 | 0.34 | 1499 |

**Table S5:** *Overview of Data Distribution Across Different Scenarios.*

| Category | Scenario | Training Images | Validation Images |
|---|---|---|---|
| Main Scenario | I | 45 | 5 |
| | II | 63 | 7 |
| | III | 63 | 7 |
| LPIPS Validation Scenario | I | 77 | 8 |
| | II | 147 | 16 |
| | III | 103 | 11 |
| | IV | 115 | 12 |
| | V | 203 | 22 |

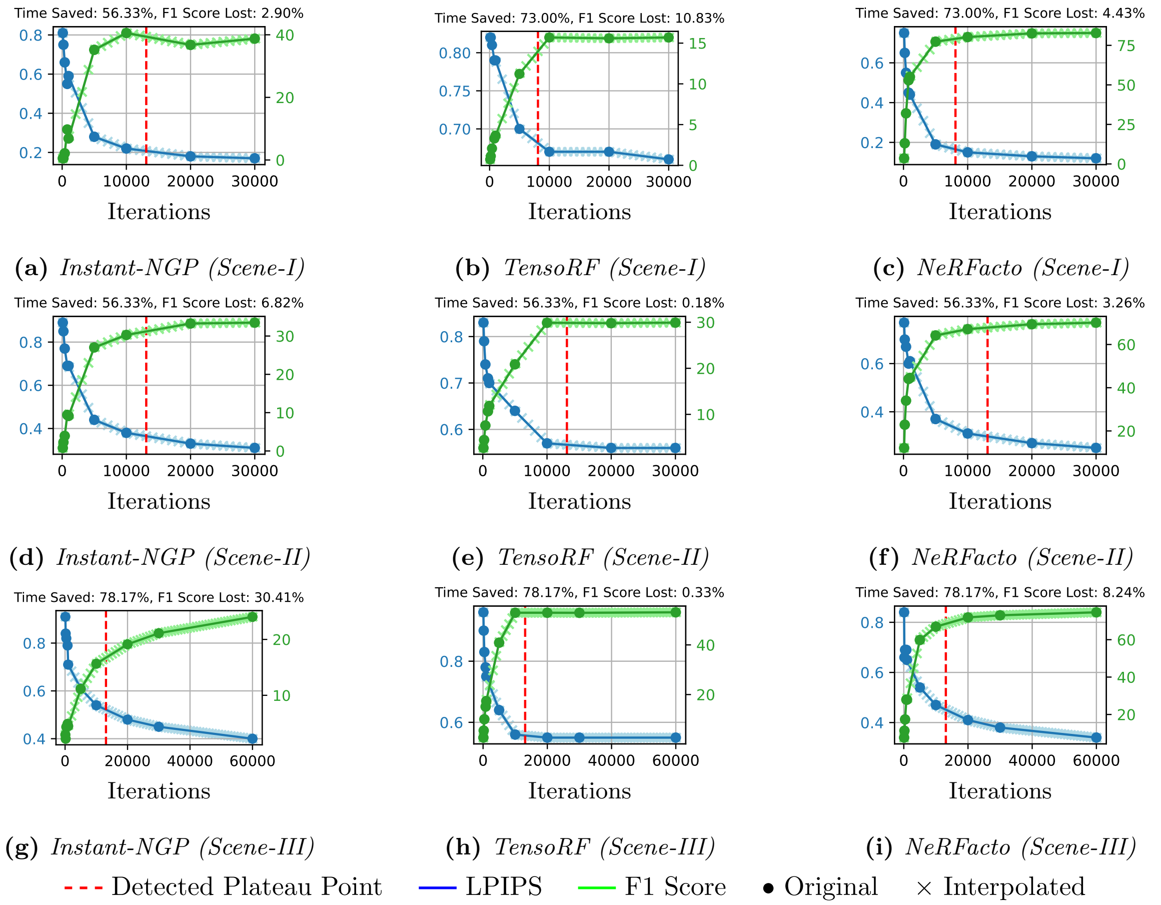


**(a)** *Instant-NGP (Scene-I)* **(b)** *TensoRF (Scene-I)* **(c)** *NeRFacto (Scene-I)*

**(d)** *Instant-NGP (Scene-II)* **(e)** *TensoRF (Scene-II)* **(f)** *NeRFacto (Scene-II)*

**(g)** *Instant-NGP (Scene-III)* **(h)** *TensoRF (Scene-III)* **(i)** *NeRFacto (Scene-III)*

**Figure S7:** *Performance of early stopping algorithm based on LPIPS on scenes with ground truth.*

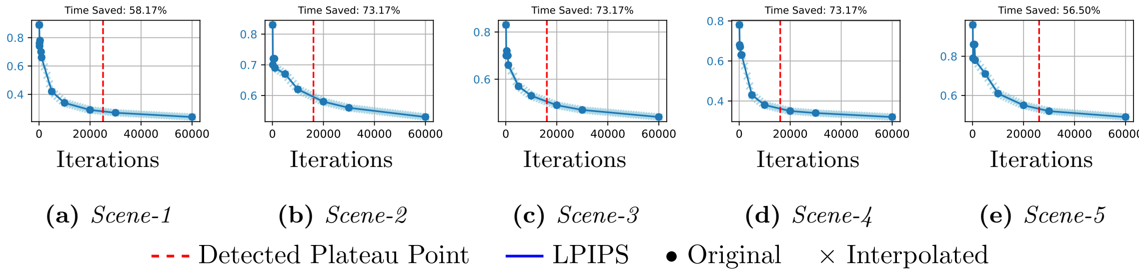


**(a)** *Scene-1* **(b)** *Scene-2* **(c)** *Scene-3* **(d)** *Scene-4* **(e)** *Scene-5*

**Figure S8:** *LPIPS on validation scenes and proposed early stoping of training.*

### S2.5 Accurate Scaling of NeRF Reconstructions

The effectiveness of using known reference objects for scaling NeRF reconstructions is demonstrated in Figure S9. We placed three calibration spheres of known dimensions alongside a plant specimen. The scene was captured using both TLS and reconstructed using NeRF. Sphere measurements from TLS served as the ground truth, with radii of approximately 69.0, 68.6, and 69.2 mm. Using the average sphere size from TLS as a reference, we scaled the NeRF reconstruction. The NeRF-derived sphere measurements were 68.6, 68.8, and 69.2 mm, showing close agreement with the TLS data. We measured the plant height to validate the scaling accuracy. The NeRF-based measurement yielded a height of 772 mm, which aligns well with manual measurements of 770 $\pm$ 5 mm (averaged over three repetitions). This example demonstrates that NeRF reconstruction, when appropriately scaled using known reference objects, can achieve dimensional accuracy within 1% of the physical measurements.

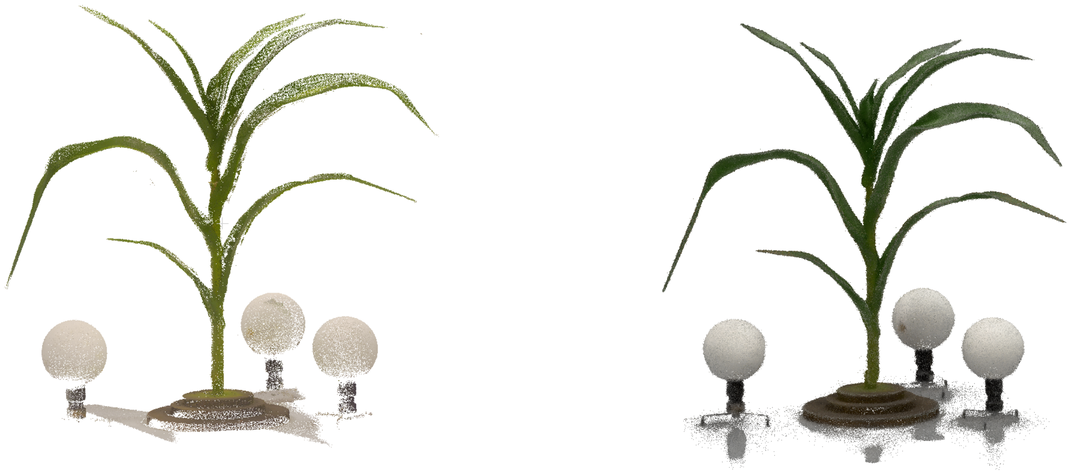

**(a)** *Original TLS* **(b)** *Scaling using NeRF.*

**Figure S9:** *Scaling comparison of 3D scene reconstruction. (a) Scene captured using Terrestrial Laser Scanning (TLS). (b) Scene reconstructed using Neural Radiance Fields (NeRF) and scaled using the calibration spheres. The plant height from the scaled NeRF aligns well with manual measurements, demonstrating NeRF's accuracy in scene reconstruction and scaling using reference objects. The plant height is measured from the stalk base to the highest point.*
